# Wildfires in Bamboo-Dominated Amazonian Forest: Impacts on Above-Ground Biomass and Biodiversity

**DOI:** 10.1371/journal.pone.0033373

**Published:** 2012-03-09

**Authors:** Jos Barlow, Juliana M. Silveira, Luiz A. M. Mestre, Rafael B. Andrade, Gabriela Camacho D'Andrea, Julio Louzada, Fernando Z. Vaz-de-Mello, Izaya Numata, Sébastien Lacau, Mark A. Cochrane

**Affiliations:** 1 Lancaster Environment Centre, Lancaster University, Lancaster, Lancashire, United Kingdom; 2 Departamento de Biologia, Universidade Federal de Lavras-UFLA, Lavras, Minas Gerais, Brazil; 3 Geographic Information Science Center of Excellence, South Dakota State University, Brookings, South Dakota, United States of America; 4 Departamento de Biologia, Universidade Federal do Paraná, Palotina, Paraná, Brazil; 5 Departamento de Biologia e Zoologia, Universidade Federal do Mato Grosso, Cuiabá, Mato Grosso, Brazil; 6 Departamento de Estudos Basicos e Instrumentais, Universidade Estadual do Sudoeste da Bahia, Itapetinga, Bahia, Brazil; DOE Pacific Northwest National Laboratory, United States of America

## Abstract

Fire has become an increasingly important disturbance event in south-western Amazonia. We conducted the first assessment of the ecological impacts of these wildfires in 2008, sampling forest structure and biodiversity along twelve 500 m transects in the Chico Mendes Extractive Reserve, Acre, Brazil. Six transects were placed in unburned forests and six were in forests that burned during a series of forest fires that occurred from August to October 2005. Normalized Burn Ratio (NBR) calculations, based on Landsat reflectance data, indicate that all transects were similar prior to the fires. We sampled understorey and canopy vegetation, birds using both mist nets and point counts, coprophagous dung beetles and the leaf-litter ant fauna. Fire had limited influence upon either faunal or floral species richness or community structure responses, and stems <10 cm DBH were the only group to show highly significant (p = 0.001) community turnover in burned forests. Mean aboveground live biomass was statistically indistinguishable in the unburned and burned plots, although there was a significant increase in the total abundance of dead stems in burned plots. Comparisons with previous studies suggest that wildfires had much less effect upon forest structure and biodiversity in these south-western Amazonian forests than in central and eastern Amazonia, where most fire research has been undertaken to date. We discuss potential reasons for the apparent greater resilience of our study plots to wildfire, examining the role of fire intensity, bamboo dominance, background rates of disturbance, landscape and soil conditions.

## Introduction

Understorey wildfires in humid tropical forests have increased in frequency and prevalence over the last three decades, mainly due to their interaction between fire-dependent agricultural practices and extreme drought events [1], [2], [3], [4]. The ecological impacts of these fires have been described in several different tropical forests [5], reporting high rates of tree mortality of up to 50% [6], [7] and strong negative impacts on forest biodiversity; most notably on understorey birds [8], [9], [10], large vertebrates [11] and leaf litter arthropods [12]. Within South America, most of these studies have been conducted in forests in the central and eastern Brazilian Amazon, following fires associated with El Niño-related drought events [3], [6], or in Bolivian forests that were logged as well as burned [13].

Much less is known about the responses of south western Amazonian forests to fire disturbance, although a large extent of forest was recently affected by severe drought events that occurred in 2005 [14] and 2010 [15]. These droughts are related to higher North Atlantic sea-surface temperatures [14], and are distinct from the El Niño associated droughts affecting the northern and eastern Amazon [16]. Conservative estimates suggest that c. 2,800 km^2^ of forest burned in the state of Acre in 2005 [17].

There are good reasons to believe that forests in western Amazonia may respond differently to fire disturbance than forests in the east. The Amazon spans a variety of different gradients, including rainfall and geological history [18], [19], and forests in western Amazonia are typically the most species rich [18], have a high turnover of stems [20], high above-ground coarse wood productivity [21], and one of the lowest average wood densities [22]. Importantly, there are suggestions that western Amazonian forests could be more resilient to disturbance than central and eastern Amazon forests. First, while trees suffer very high edge-related mortality rates in central Amazonian forests [23], a similar loss of aboveground biomass was not observed near forest edges in south-western Amazonian forests [24]. Second, eastern Amazonian forests may contain more senescent large trees than western Amazon forest [25], and these individuals could be more vulnerable to the physiological stresses associated with any additional disturbance such as fire (e.g. [26]).

In addition, some areas of south-western Amazonia may have a long historical association with fire disturbance. Around 165,000 km^2^ of forests shared by Brazil, Peru and Bolivia are dominated by *Guadua* bamboos [27]. Although these forests cover only a small fraction of the total areal extent of the Amazon (c. 2%), they cover twice as much land as the remaining forest cover in Brazil's Atlantic Forest [28]. There are three reasons why these bamboo-dominated forests could respond differently to fire disturbance. First, the presence of bamboo suggests that fire may have played an important role in shaping these forests [27]. Second, *Guadua* bamboos appear to have a limited ability to withstand drought, and may increase forest flammability as they shed leaves after only a few days without rain [27]. Third, the synchronous dieback of semelparous *Guadua* could increase forest flammability [29].

We conducted the first assessment of the ecological impacts of the 2005 wildfires in bamboo-dominated forests in the Brazilian Amazon. We compared biodiversity and forest structure in plots in burned and unburned forests that were sampled three years after the fires took place, and compared change in forest structure with data from burned forests in the central Brazilian Amazon. Specifically, we examined the following research questions: 1) what was the impact of fire on forest structure and the richness and composition of forest biodiversity? 2) How do these changes compare with those recorded in the central and eastern Brazilian Amazon? We also assessed to what extent potential factors (such as drought-related mortality, [30], disturbance history, and fire intensity) could explain these differences.

## Methods

### Study area

This study was conducted in the “RESEX Chico Mendes” (10° to 11° S, 68° to 70° W), in the Brazilian state of Acre in southwestern Amazonia, which receives around 2200 mm a year [31]. The RESEX covers approximately 1 million hectares of *terra firme* forests on mostly clay-dominated soils. These forests contain a high density of two species of semi-scandent woody bamboos, *Guadua sarcocarpa* Londoño & P.M. Peterson and *G. weberbaueri* Pilg. [27]. These results were compared with data published in studies from the central Brazilian Amazon in the state of Pará [6], [9].

### Sampling methods

**Figure 1:**
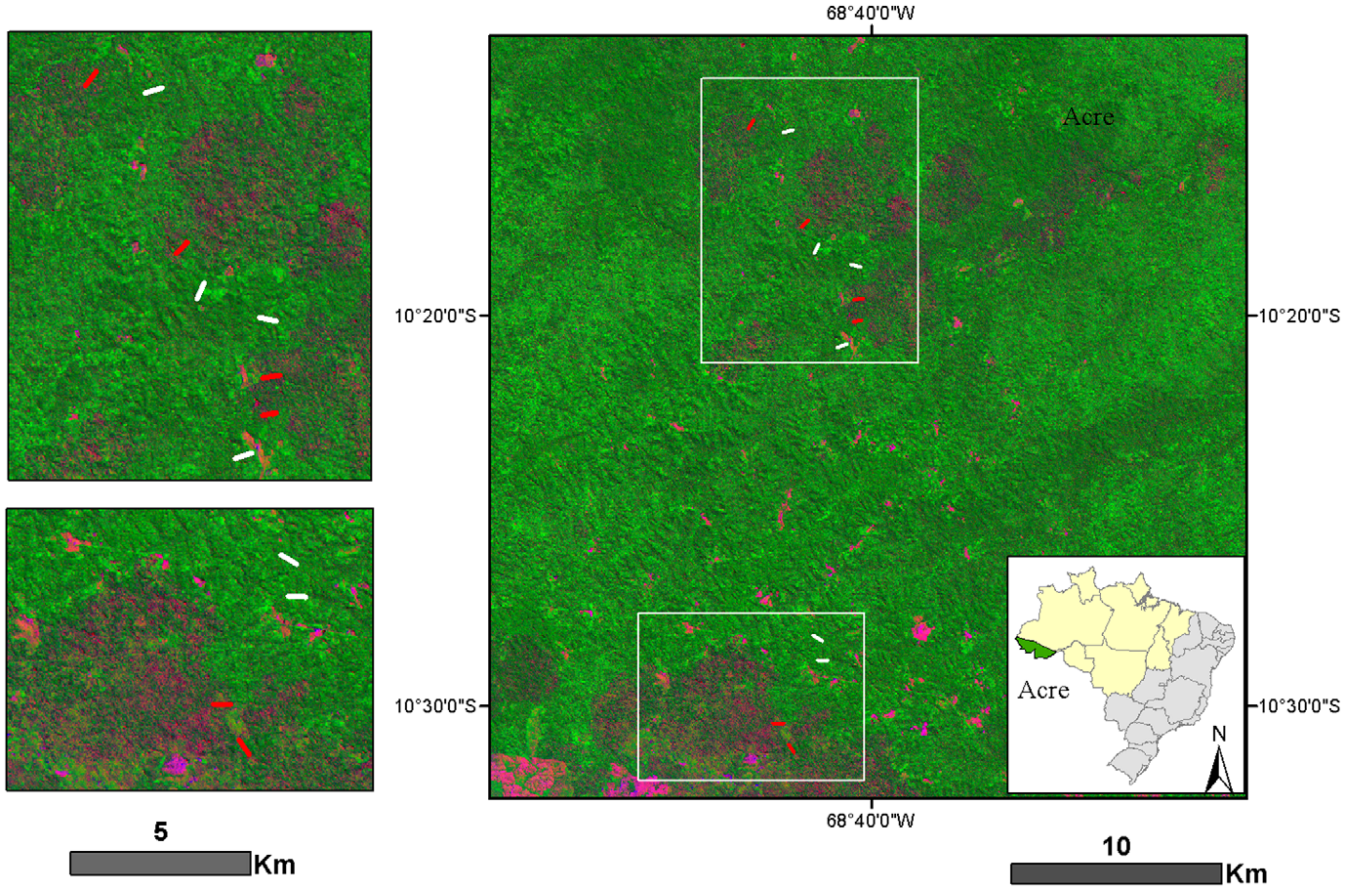
Landsat imagery of the study area (26/062006), showing fire scars (blotchy red areas) from the 2005 fires and the location of the 6 burned and 6 unburned study plots in the RESEX Chico Mendes (red and white bars, respectively).

**Figure 2:**
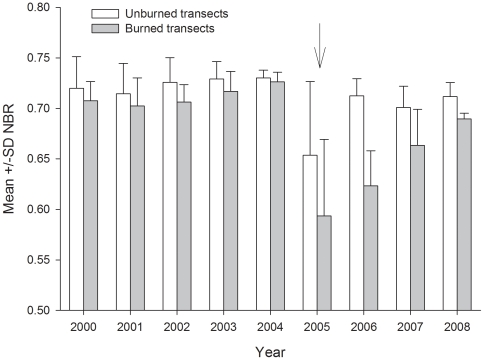
Mean ± SD of the Normalized Burn Ratio (NBR) across the six unburned and six transects that burned in 2005 (arrow). The NBR is widely used to detect burned forests from Landsat reflectance data. The lower NBR values in unburned forests in 2005 likely reflect smoke from ongoing fires (the image was taken on the 11^th^ of September during the peak of the fire season) and possibly some impact of the drought. The NBR values prior to 2005 provide evidence suggesting the transects were similar before the fires; the NBR values after 2005 show the stability of the unburned plots, and the recovery of canopy cover in the burned plots, up to the time of the field sampling in 2008.

We sampled forest structure and biodiversity along twelve 500 m transects between September and November 2008 (Figure 1). Six transects were placed in unburned forests and six were in forests burned during a series of forest fires that occurred within the RESEX from August to October 2005. All transects were located at least 1 km from each other, and were positioned without *a priori* information on the severity of the fires or forest composition. An analysis of Normalized Burn Ratio (NBR) values [32] derived from annual Landsat imagery for five years prior to (2000–2004) and three years after the fires (2006–2008) revealed similar spectral reflectance patterns in burned and unburned plots before the fires (Figure 2).

We sampled all trees ≥10 cm DBH and all lianas ≥5 cm DBH in 10×500 m (0.5ha) vegetation plots laid out to the side of each transect. Stems were classified as dead if none of the bark at breast height was living (implying the entire above-ground biomass associated with that stem was dead). However, some of these same individuals exhibited basal resprouting, which we also recorded. For smaller stems <10 cm DBH, we sampled two 5×5 m subplots at four points, located at 50 m, 200 m, 350 m and 500 m, along each transect, with plots located on either side and one metre from the transect (avoiding any disturbance that may have occurred when the transects were opened). The diameter of smaller stems was measured with dial calipers. We also sampled canopy openness at each point using hemispherical photographs (analysed with Gap Light Analyzer http://www.ecostudies.org/gla/), and measured leaf litter volume by placing 0.25 m^2^ quadrats of leaf litter into a 26 cm×28 cm cylinder (the Winkler extractor used for the ants). To measure leaf litter (and collect ants) we used four quadrats per point, each spaced 3 meters from the central point.

For comparative purposes, we contrasted data on trees ≥10 cm DBH in Acre with data collected in or around the RESEX Tapajos-Arapiuns in Pará in the central Brazilian Amazon (2°44°S, 55°41°W), located c. 1600 km east of the Acre study sites. Sixteen 0.25ha plots in unburned forest and 22 0.25ha plots in burned forest were sampled in 2000–2001, three years after wildfires associated with the 1998–1999 El Niño event (See [6] for more details). We also reanalysed data on the community turnover of birds captured in mist nets in 12 burned and ten unburned plots in Pará [9], to provide a comparative basis for the bird data in Acre (see SOM).

Faunal sampling in Acre occurred at the same four sample points along each transect. Ants were sampled using the Winkler methodology, using the same quadrats used to collect leaf litter volume data. After registering the leaf litter volume, each one of the four quadrats per point was sieved (mesh = 1 cm^2^) to remove larger debris and leaves, and combined into a single sample. The fine material was then placed into Winkler extraction funnels that were suspended in the shade for 72 hours. Ants were sorted in a laboratory and identified by an expert ant taxonomist (S. Lacau). Voucher specimens are stored at the Universidade Estadual do Sudoeste da Bahia-UESB, Itapetinga, Bahia, Brazil.

Dung beetles were sampled using pitfall traps baited with human feces [33], [34]. Each pitfall consisted of a cylindrical plastic container (15 cm wide, 9.5 cm deep) buried at ground level and quarter-filled with salted water and a drop of detergent. A small bag made of cotton gauze containing c. 25 g of human feces was suspended above the pitfall. The lid of the plastic container was placed 10 cm above the trap with three wooden sticks, helping protect both the bait and the pitfall from rain. All traps were collected after 48 hours, rebaited and collected again after a further 48 hours. All specimens were processed at the Universidade Federal de Lavras with identifications confirmed at Universidade Federal de Mato Grosso. Voucher specimens are deposited in both institutions.

Birds were sampled using both mist-nets and point counts. Twenty eight mist nets (12×2.5 m; mesh size 36 mm) were erected in four groups of seven nets. Each group created a netline of 7×12 m extending for 90–100 m. Groups were separated by an open space of 50 m. During two days, we opened the 28 nets from 0630h (sunrise) to 1330 h, totaling 4704 mist-net hours. We checked nets hourly and closed them during periods of heavy rain. All captures were identified to species, weighed, measured (standard measurements included wing, tail, bill, and total length) and, whenever possible, were aged, sexed and photographed. They were also banded with a numbered metal ring obtained from Centro Nacional de Pesquisa para Conservação de Aves Silvestres (CEMAVE) - Instituto Chico Mendes de Conservação e Biodiversidade (ICMBio). All recaptures from the same sampling period and from the same net line were excluded from the analysis. Ten minute point counts were carried out twice at each sampling point, on two different days and avoiding transects where mist-netting was taking place. The repeat visits included an early sample (0600–0730 h) and a later sample (0730–0900 h). We used a digital recorder and a directional microphone to record all acoustic registrations, and unknown vocalizations were subsequently checked against known calls. Distance from the observer and height were also noted. To ensure independence between point-counts, we excluded birds flying over the canopy and any registrations >50 m from each point count. To avoid double counting during the second visit, we excluded any observations of species that had already been recorded at that point. We analyzed mist net and point count data separately, as the two provide complementary information about forest bird communities [35].

### Ethics statement

We are very thankful to the Instituto Chico Mendes para a Conservação da Biodiversidade- ICMBio and the communities of RESEX Chico Mendes for their permission and help with the field work. All necessary permits were obtained for the described field studies, including permission to collect ants and dung beetles within the Chico Mendes Extractive Reserve (Sisbio permit no. 178811-1) and authorization for ringing birds (project no. 3012/4 from CEMAVE/Ibama). Plants were identified in situ by an expert parataxonomist.

### Statistical analysis

Patterns of species richness between different unburned and burned forests were compared using an individual-based rarefaction procedure within EstimateS (v.7) [36], where individuals are set as samples and the curves are then calculated using the Mao Tao estimator. Significant differences between habitats were assessed by visual inspection of 95% confidence intervals. Significance at p<0.05 may be assessed by observing whether the burned forest curve lies within the 95% confidence interval of the curve of the unburned forest [37].

Changes in community structure were assessed using ordination analyses implemented in R 2.14.0 and Primer v. 6, using non-metric multi-dimensional scaling (MDS) based on the Raup-Crick disimilarity metric. The Raup-Crick metric calculates the probability that the compared sampling units have non-identical species composition, and is considered the most appropriate metric for sparse datasets with many absences, which are typical of species rich tropical communities [38], [39]. It was calculated in the “vegan” package in R 2.14.0 using the “raupcrick” command. The influence of fire on community structure and community dispersal were examined using Permanova and Permadisp, respectively, conducted in Primer v.6. A randomization test (Indicator Species Analysis [40]) was used to examine which plant species were significantly more abundant and frequent in unburned or burned forest. IndVal analysis was undertaken in PCORD [41]. Comparisons of forest structure used a One-Way Permutation Test (with 9999 Monte-Carlo resamplings) conducted in the Coin package in R 2.12.2. [42].

Considering the large number of statistical tests conducted in this manuscript, we consider p<0.05 as providing marginal support, and p<0.01 as providing strong support. We did not adopt a more stringent correction (such as a Bonferroni correction) as this would merely increase the chance of Type II errors, especially given our relatively small sample size.

## Results

In total, we measured 2685 stems ≥10 cm DBH and 3672 stems <10 cm DBH in our plots in the south-western Amazon. We also captured 3492 dung beetles, recorded 603 ant occurrences, and sampled 868 birds in mist-nets and 1586 in point counts (excluding those >50 m from the observer or flying over). We examine changes in forest structure, compare the data with burned forests in Pará, and then assess how fire altered the species richness and community structure of the studied *taxa*.

### Changes in forest structure

**Figure 3:**
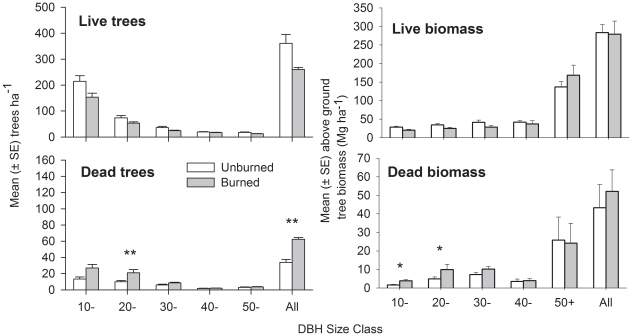
Mean (±SE) number of trees and mean (±SE) aboveground biomass per hectare, measured three years after fire. Significant differences between unburned and burned plots are shown by *<0.05, **<0.01.

The number of live trees ≥10 cm DBH and mean aboveground live biomass were statistically similar in the unburned and burned plots (Figure 3). There was a significant increase in the number of standing dead trees overall, and in the 20–20.9 cm DBH size class (p<0.01). Total aboveground dead and live biomass was not significantly different in unburned and burned plots, but there were marginally significant increases in standing dead biomass in the 10–19.9 and 20–29.9 cm DBH size classes (p<0.05, Figure 3).

We measured 122 lianas ≥5 cm DBH across the twelve 0.5 ha plots, of which only 25% were ≥10 cm DBH. The data indicate a high mortality rate of lianas in these forests, with mean (± SE) density falling from 32.0±3.2 ha^−1^ in unburned forest to 8.7±2.6 ha^−1^ in burned forests (Table 1). Dead lianas did not appear to persist in the burned forests, and we only recorded one dead liana stem in the unburned control plots. Burned plots also had a marginally significant higher proportion of resprouting trees ≥10 cm DBH and a more open canopy than the control plots (Table 1). Other forest structure variables were non-significant (Table 1).

**Table 1:** Average values for forest structure variables measured in burned (BF) and unburned (UF) forests in Acre.

	Units	Mean UF	SE	Mean BF	SE	Z	P
*Data from 0.5 ha plots*							
Lianas	Per 0.5ha plot	16.0	1.6	4.3	1.2	−2.88	0.002**
Palms 10–19.9	Per 0.5ha plot	31.3	6.9	37.7	19.0	−0.80	0.49
Palms 20+	Per 0.5ha plot	22.0	7.5	20.3	5.4	−0.16	0.94
Dead palms	Per 0.5ha plot	4.7	1.2	7.8	3.8	−2.41	0.82
Canopy intact	(%)	90.4	3.9	88.0	2.6	−1.12	0.31
Stems resprouting	(%)	0.4	0.1	2.2	0.6	−2.01	0.04*
*Data from subplots*							
Stems <2.5 cm DBH	Per transect (200 m^2^)	22.7	2.3	17.8	1.8	−0.70	0.65
Stems 2.5–4.9 cm DBH	Per transect (200 m^2^)	168.8	36.4	144.2	9.0	0.68	0.54
Stems 5.0–9.9 cm DBH	Per transect (200 m^2^)	112.8	24.0	95.5	8.3	−1.52	0.15
All live stems <10 cm DBH	Per transect (200 m^2^)	279.7	28.8	282.2	30.7	0.06	0.95
*Structure data*							
Leaf litter volume	Volume (m^3^ per m^2^)	0.023	0.001	0.018	0.007	−2.0	0.04*
Canopy openness	(%)	20.0	1.48	24.5	1.25	2.1	0.03*

Significance was tested using one-way permutation tests.

P values are *<0.05, **<0.01, ***<0.001.

### Comparing fire severity in Acre and Pará

**Figure 4:**
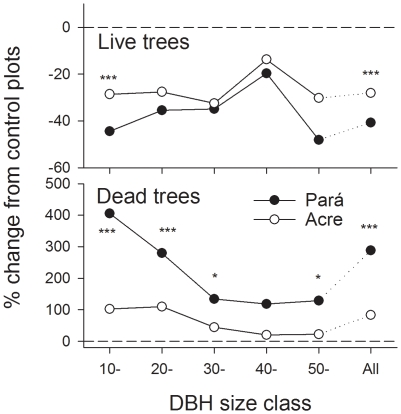
The percentage change in the number of live and dead trees recorded three years after wildfires in the RESEX Arapiuns-Tapajos (Pará) and the RESEX Chico Mendes (Acre). Horizontal dashed lines represent the average values recorded in control plots in each region. Significant differences were tested with chi-square, using the % change in Para to calculate the expected change in Acre. Significance is shown as *<0.05, **<0.01, ***<0.001.

Our comparisons of the percentage change in the number of live and dead trees three years after wildfires suggest that fire severity (measured as its impact on trees ≥10 cm DBH) was lower in the south-western Amazon plots than those in the central Amazon (Figure 4). In every DBH size class, the difference between the numbers of live stems in burned and unburned forests were numerically smaller in Acre than they were in Pará. These numerical differences were supported by statistical comparisons of dead stems ≥10 cm DBH, which were 288% more abundant in burned forests in Pará, but only 83% more abundant in burned forests in Acre (p<0.001, Figure 4).

### Impacts of fire on biodiversity

#### Species richess

**Figure 5:**
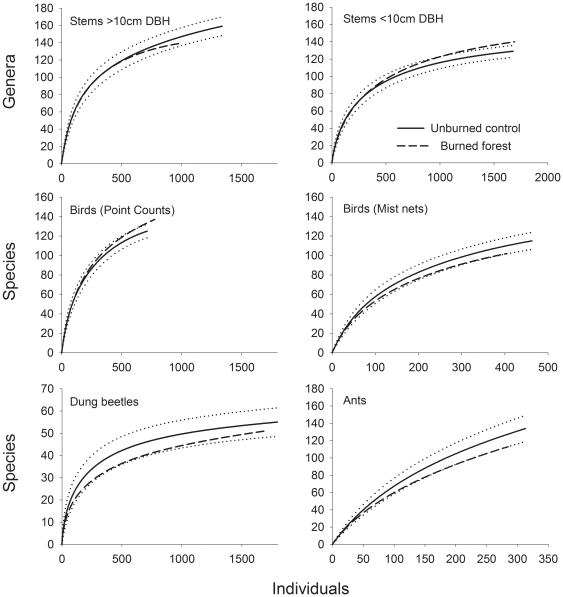
MDS ordination and Analysis of Similarity results comparing community structure in unburned control plots (clear symbols) and burned forest plots (grey symbols).

Our surveys demonstrate the very high species richness of bamboo-dominated forests in the south-western Amazon (Figure 5). Species acumulation curves suggest many species or genera remain unsampled, especially for the ants and birds. Fire had a mostly insignificant influence on faunal species richness, either when data was pooled (Figure 5) or when comparing species richness at the transect scale (Figure S1). There is a suggestion that fire disturbance reduced the richness of plant genera across the 0.5ha plots, and stems <10 cm DBH appear to be significantly more species rich in the burned forests. Neither of these patterns were observed at the transect scale (Figure S1).

#### Species composition and community structure

**Figure 6:**
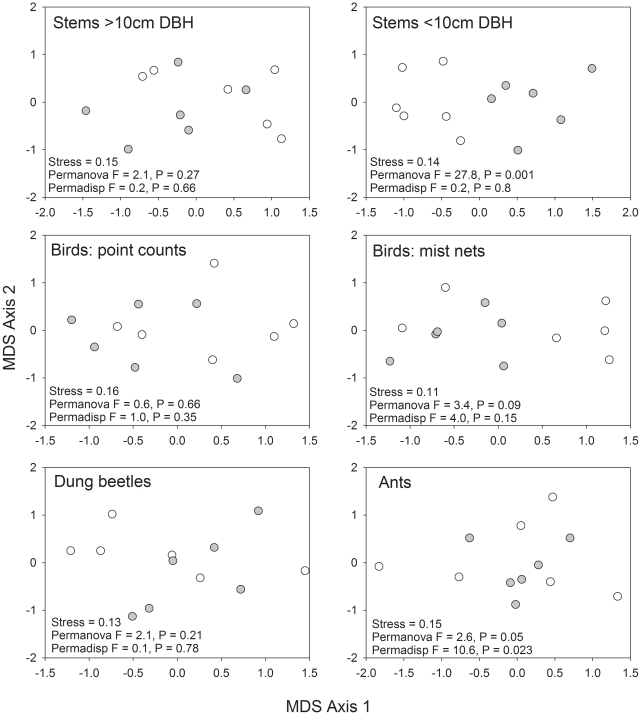
Individual-based species accumulation curves comparing species richness in burned forest plots with the unburned control plots. The dotted lines represent the 95% confidence interval for the unburned forests.

There were mixed community responses to fire disturbance across the *taxa* we sampled (Figure 6). Within the vegetation, there was no significant difference in the species composition of stems ≥10 cm DBH, but the species composition of stems <10 cm DBH was significantly different between burned and control forests (Figure 6). Only a few genera of stems <10 cm DBH showed significant responses in their abundance, with a marginally significant loss of the genera *Tachigalia* and *Eugenia* from the burned sites (Figure S2). Five genera were significantly (*Urera* and *Actinostemom*; p<0.01) or marginally significantly (*Zanthoxilon*, *Sapium*, *Apeiba*; p>0.05) more abundant after fire. *Guadua* bamboos almost doubled in abundance in burned forests, but these differences were not significant.

Within the fauna, only leaf-litter ants showed a difference in community structure, which was marginally significant for community turnover and community dispersal (p<0.05; Figure 6). Their were no significant difference for the dung beetles or the bird communities sampled by mist nets or point counts (Figure 6).

#### Checking the validity of our analyses

We undertook a series of analyses to ensure our space-for-time assessments of the consequences of wildfires were valid in Acre. The 10 year comparative assessment of Normalized Burn Ratio (NBR) values across the six unburned and six transects that burned in 2005 did not suggest that the transects were consistently different before the fires (Figure 2). As fire is a highly spatially auto-correlated disturbance event [43], we also examined whether our results could be explained by the spatial layout of the sampling transects. Again, we found no evidence to suggest this was the case, and all RELATE correlations between community structure and the geographic distance between sites were insignificant (Stems ≥10 cm DBH, Rho = −0.31, p = 0.97; Stems <10 cm DBH, Rho = −0.08, p = 0.73; Birds (point counts) Rho = −0.25, p = 0.96; Birds (mist nets) Rho = −0.07, p = 0.73; Ants, Rho = 0.03, p = 0.38; Dung Beetles Rho = −0.55, p = 1.0).

We also investigated potential confounding factors that could explain the different effects of fires in Pará and Acre. First, we evaluated fire intensity by comparing our measurements of char height from both regions. These suggest that fires were more intense (had higher average flame heights) in Acre. Out of 1214 stems measured in burned forest in Acre, 544 showed scars or charring that could be related to fires, and 303 (25%) showed some kind of charring ≥1 m in height. This compares with just 7% of stems that showed scars or charring ≥1.3 m three years after the fires in Pará [44]. Second, we examined factors that could influence fire intensity in unburned plots in Acre and Pará. Again, these suggest that fires were likely to be more intense in Acre, where the unburned forests had a significantly more open canopy, and a greater volume of leaf litter on the forest floor (Figure S3).

The impact of fires on tree mortality in the smaller size classes could be masked by the rapid regeneration of stems three years after fire in Acre. As it was difficult to make accurate *in situ* assessments of whether a stem had regenerated before or after the fire, we used a statistical approach examining community turnover in stems in the 10–19.9 cm DBH range, with the expectation that high community turnover between burned and unburned forest in this size class would provide evidence of a significant recruitment of pioneers. The test was not significant (Permanova Pseudo-F = 1.37, p = 0.33), and the lack of regenerating pioneers in this 10–19.9 cm DBH size class is further demonstrated by comparing the abundance of fast-growing pioneers such as *Cecropia* spp., as we recorded 9 stems in unburned forests and 14 in burned forest.

To examine the potential impact of drought related mortality, we compared the overall number of standing dead stems per hectare in our unburned forest controls in Acre and Pará. These numbers were identical (Figure S4). We also compared the genus richness of stems ≥10 cm DBH in the unburned forests of Acre and Pará, which was not significant (Figure S3). Finally, as differences in selective logging could have had a big influence on both the likelihood and intensity of fires [3], [4], [45], it is important to clarify that neither region had undergone commercial timber extraction.

## Discussion

We provide a first assessment of the impacts of forest wildfires in south-western Amazonia. Some of our findings are consistent with those from other regions of the Amazon: the overall pattern of tree mortality across size classes was visually similar to that found in burned forests elsewhere in the Amazon and stems in the 40–40.9 cm size class were least affected by fire disturbance (Figure 4), mortality of large diameter woody vines was very high [7], [46], [47], and palm trees are apparently able to survive fires [11].

However, our data also suggest that wildfires had much less effect upon forest structure and biodiversity in these study plots than in central and eastern Amazonia, where most fire research has been undertaken to date. Tree mortality was higher across all stem size classes in plots in the central Amazon than in south-western Amazonia, and these differences were highly significant in the 10–19.9 cm size class (Figure 3). Furthermore, the fires in Acre had a relatively minor influence on the turnover of birds captured in mist nets in Acre (Figure 6), when compared to the highly significant turnover in bird species composition observed in the understorey bird community in the central Amazon [9] where there was no overlap between the composition of burned and unburned plots (Figure S5), or dung beetles in the southern Amazon [34]. We considered several complementary lines of evidence that could indicate why the impacts of fire were less severe in south-western Amazonian forests than in central and eastern Amazonia. As we have no direct evidence for these, we phrase them as hypotheses that can be tested and considered in future work.

### 1. Fire impacts are related to fire intensity

The most obvious explanation for differences in the impact of fires relates to the behaviour of the fires themselves. However, our results are counter-intuitive, as many lines of evidence suggest that the fires in the forests we studied in Acre were more intense than those in Pará. First conditions in unburned forests in the region should indicate more intense fires, as these forests had more open canopies (reducing understorey humidity [48]), deeper leaf litter (increasing the fine-fuel loading; Figure S3), and a high abundance of pyrophytic bamboos that are absent from primary forests in Pará. Second, local residents of the RESEX Chico Mendes reported intense fires that occasionally reached into the canopy (J.Silveira pers. obs.), while wildfires reported and observed in forests in central and eastern Pará were mostly slow-moving understorey fires with flame heights that rarely exceeded 30 cm [3], [6]. Third, these qualitative observations were also confirmed by our measurements of char height on stems (see Results). As flame height is a significant predictor of tree mortality [49], [50], this cannot explain the lower tree mortality in Acre. However, we are unable to rule out flame residence times, which may have been lower in Acre if fires were fast moving, and this would be an important area of study in the future.

### 2. Fire impacts are related to recent disturbance history or drought sensitivity

Variation in fire-induced tree mortality could relate to the number of times a forest has burned [3], the history of timber extraction [4], [45], time since the last fire event, or possibly interactions with simultaneous drought-induced mortality [30]. However, we believe that these can be discounted in our comparison of plots in central and south-western Amazonia: both sites were examined three years after the fires, neither site appears to have been burned before or undergone mechanised timber extraction, Landsat images did not reveal any consistent differences in forest reflectance before the fires (Figure 2), and there was no apparent difference in drought sensitivity in the unburned plots (Figure S4).

### 3. The lower impact of fires in Acre is a result of landscape scale effects

The size and spatial extent of fire has consequences for fire impacts and subsequent regeneration [51]. Although we placed our transects randomly in the landscape, and without *a priori* knowledge of burn extent, by chance many of the transects were close to the adjacent unburned areas in Acre (Figure 1). As this did not seem to reduce burn intensity (see hypothesis 1), it seems unlikely we measured transects that were affected by the onset of the rains that finally extinguished the fires, and the spatial positioning of our transects does not explain the low rates of tree mortality. However, the close proximity and favourable landscape context may have provided ample source populations for recolononising burned areas, and could go some way to explaining the minimal impact on faunal populations. However, previous assessments of edge related effects suggest the fireline acts as an abrupt barrier, at least to understorey birds one year after fires in the central Brazilian Amazon [52]. More work on post-fire forest recovery and the importance of source populations is required .

### 4. A prolonged history of disturbance in bamboo-dominated tropical forests has acted as an extinction filter or a selective force, extirpating species that are most sensitive to fire and encouraging local adaptations to disturbance among the remaining species

Bamboo-dominated tropical forests are affected by large-scale disturbance events, both during the synchronous flowering and dieback of bamboos, and because the pyrophytic vegetation can encourage wildfires [27]. This latter argument is supported by evidence from charcoal records, which suggests that some of the more seasonal locations of the Amazon have a long fire history from 8000 to 4000 years B.P [53]. However, the comparable richness of tree and liana genera along transects in Acre and Pará (See Figure S3) does not suggest that these disturbance events have imposed a strong extinction filter on the flora of Acre. Overall, the influence of past-fires on large-scale patterns of plant species diversity and composition found in the Amazon basin is poorly known, but certainly requires further consideration [54].

### 5. The faster growth and turnover of trees in south-western Amazonian forests engenders greater resilience to fire-disturbance

Trees in south-western Amazonian forests grow and die faster than those in the central and eastern Amazon [20], [21], meaning disturbance events (in the form of tree falls) are inherently more frequent. This is in part reflected by the more open canopy in south-western Amazonian forests (20% in unburned forests in Acre compared to 12.5% in Pará; Figure S3). The relatively more open canopy and denser understorey of these forests could make the flora and fauna more resilient to the structural changes that take place after fires, and would explain the relative resilience of the understorey birds in south-western Amazonian forest (Figure 6) when compare to the more drastic turnover in species composition that occurred after wildfires in Pará [9]. While we do not have any data to test this, it makes intuitive sense that species inhabiting forests that are disturbed more frequently will become adapted to those disturbance events if they take place over a long enough period of time.

### 6. Large trees respond differently to disturbance in eastern and western Amazonian forests

There is some evidence to suggest that the drivers of large-tree mortality are fundamentally different across the Amazon, as north-eastern Amazonian forests contain more senescent large trees than those in the north-western Amazon [25]. Assuming this east-west difference is consistent, and senescent individuals are more vulnerable to additional physiological stress associated with disturbance (such as fire), then we would expect the large-trees in south-western Amazonian forests to be more resilient to fire than those in eastern Amazonian forests. This expectation provides a close match with our observed results of high rates of large-tree mortality in eastern Amazonia [26] and much lower rates in south-western Amazonian forests (Figures 2 and 3). Large trees account for a highly disproportionate amount of forest biomass relative to their abundance [55] (Figure 3), and more work is needed to understand their response to forest disturbance. Given the spatially dispersed distribution of these large trees, future studies should consider using much larger plots to achieve a more representative sample of this important component of forest structure.

### 7. Soil type and structure can have an important role in determining faunal and floral mortality and post-fire regeneration

The soils of our study sites in the RESEX Chico Mendes were dominated by clay, while those in Pará were mostly located on white sands. The greater water retention capability of clay soils could affect responses to fire in two ways. First, they could enhance resilience, as clay soils are likely to help trees maintain their leaf cover during drought events. Although this does not appear to have reduced fire intensity, it may have reduced mortality if drought-stressed trees are also more susceptible to fire stress. Second, they could aggravate the impacts of fires as the greater moisture content will enhance soil heating [56], leading to a higher mortality of the seed bank [57], higher root mortality, and a greater impact on the soil fauna (including dung beetle larvae and some ants). Although we are unaware of any studies comparing tree, root and seed bank survival across different soil types, these areas deserve further investigation.

## Conclusions

These results add to the growing awareness that tropical forests respond to disturbance in different ways [58], and, in particular, the potential differences between eastern and western Amazonian forests [24], [25]. They also highlight the hitherto neglected impacts of fire in south-western Amazonian forests, and in particular those dominated by bamboos. Bamboo-dominated tropical forests are found across South America [27], [59], [60], and provide habitat for many rare and endemic species of bird [61], [62]. Increased droughts and associated wildfires could place substantial pressure on these unique systems, especially if surface fires favour increased density of bamboo culms [27]. Positive feedback dynamics involving recurrent and more frequent fires have not been studied in the forests of south-western Amazonia, but these could become increasingly important given the propensity for severe drought events in this region [14], [15] and the possible interactions with flammable bamboos [27]).

Finally, in attempting to explain our counter-intuitive results, we hope that this study raises many relevant questions about the response of Amazonian forests to disturbance, and serves to stimulate further research. Importantly, we strongly caution against applying these results from single fire events in intact forests to assume longer-term resilience in the face of continuing human pressure and a changing climate.

## Supporting Information

Figure S1 Comparisons of species richness at the transect level (n = 6 for each treatment) for all six taxa. Statistics are shown for one-way permutation tests. None of the comparisons were significant at p<0.05.
(DOC)

Figure S2 Rank abundance of plant genera for stems <10 cm DBH recorded in control plots (clear bars) and burned forest plots (grey circles). Section a) ranks the 30 most abundant genera in unburned forest, section b) shows other genera that were significantly more abundant in unburned forest than in burned forests, and section c) ranks the genera that were most abundant genera in burned forest and were not already included in sections a or b. Significance differences for burn treatments are represented by *<0.05, **<0.01.
(DOC)

Figure S3 Leaf litter depth and canopy openness and the genera richness of stems ≥10 cm DBH along transects placed in unburned forests in Acre (n = 6) and Pará (n = 4) and measured in 2008. Statistics are shown for one-way permutation tests.
(DOC)

Figure S4 The mean number of standing dead trees recorded in unburned forests in Pará and Acre. There was no significant difference in any size class, and the overall numbers were almost identical.
(DOC)

Figure S5 Community turnover in birds captured in mist nets in burned and unburned forests, three years after fire in Pará. Data are from [9], but are reanalyzed using the Raup-Crick dissimilarity metric to provide a basis for comparison with the mist net data shown in Figure 6 (main text).
(DOC)
