# Correction: Wildfires in Bamboo-Dominated Amazonian Forest: Impacts on Above-Ground Biomass and Biodiversity

**DOI:** 10.1371/annotation/8013f95e-71f4-4ec0-afe9-00cea6627685

**Published:** 2012-06-13

**Authors:** Jos Barlow, Juliana M. Silveira, Luiz A. M. Mestre, Rafael B. Andrade, Gabriela Camacho D'Andrea, Julio Louzada, Fernando Z. Vaz-de-Mello, Izaya Numata, Sébastien Lacau, Mark A. Cochrane

There was an error in the placement of the legends for Figures 5 and 6. The legends were swapped. 

