# Supplementary material for: Wildfires in Bamboo-Dominated Amazonian Forest: Impacts on Above-Ground Biomass and Biodiversity
Source: PLoS One. 2012 Mar 9;7(3):e33373. doi: 10.1371/journal.pone.0033373 (PMC3302859; doi:10.1371/journal.pone.0033373)
Supplement: Figure S1 — Comparisons of species richness at the transect level (n = 6 for each treatment) for all six taxa. Statistics are shown for one-way permutation tests. None of the comparisons were significant at p<0.05. (DOC) [file pone.0033373.s001.doc]

**Wildfires in bamboo-dominated Amazonian forest: impacts on above-ground biomass and biodiversity**

**Supporting Information Figure S1**

**Figure S1.** Comparisons of species richness at the transect level (n = 6 for each treatment) for all six *taxa*. Statistics are shown for one-way permutation tests. None of the comparisons were significant at p<0.05.
