# Supplementary material for: Wildfires in Bamboo-Dominated Amazonian Forest: Impacts on Above-Ground Biomass and Biodiversity
Source: PLoS One. 2012 Mar 9;7(3):e33373. doi: 10.1371/journal.pone.0033373 (PMC3302859; doi:10.1371/journal.pone.0033373)
Supplement: Figure S2 — Rank abundance of plant genera for stems <10 cm DBH recorded in control plots (clear bars) and burned forest plots (grey circles). Section a) ranks the 30 most abundant genera in unburned forest, section b) shows other genera that were significantly more abundant in unburned forest than in burned forests, and section c) ranks the genera that were most abundant genera in burned forest and were not already included in sections a or b. Significance differences for burn treatments are represented by *<0.05, **<0.01. (DOC) [file pone.0033373.s002.doc]

**Wildfires in bamboo-dominated Amazonian forest: impacts on above-ground biomass and biodiversity**

**Supporting Information Figure S2**

**Figure S2.** Rank abundance of plant genera for stems <10cm DBH recorded in control plots (clear bars) and burned forest plots (grey circles). Section a) ranks the 30 most abundant genera in unburned forest, section b) shows other genera that were significantly more abundant in unburned forest than in burned forests, and section c) ranks the genera that were most abundant genera in burned forest and were not already included in sections a or b. Significance differences for burn treatments are represented by * <0.05, ** <0.01
