# Supplementary material for: Wildfires in Bamboo-Dominated Amazonian Forest: Impacts on Above-Ground Biomass and Biodiversity
Source: PLoS One. 2012 Mar 9;7(3):e33373. doi: 10.1371/journal.pone.0033373 (PMC3302859; doi:10.1371/journal.pone.0033373)
Supplement: Figure S3 — Leaf litter depth and canopy openness and the genera richness of stems ≥10 cm DBH along transects placed in unburned forests in Acre (n = 6) and Pará (n = 4) and measured in 2008. Statistics are shown for one-way permutation tests. (DOC) [file pone.0033373.s003.doc]

**Wildfires in bamboo-dominated Amazonian forest: impacts on above-ground biomass and biodiversity**

**Supporting Information Figure S3**


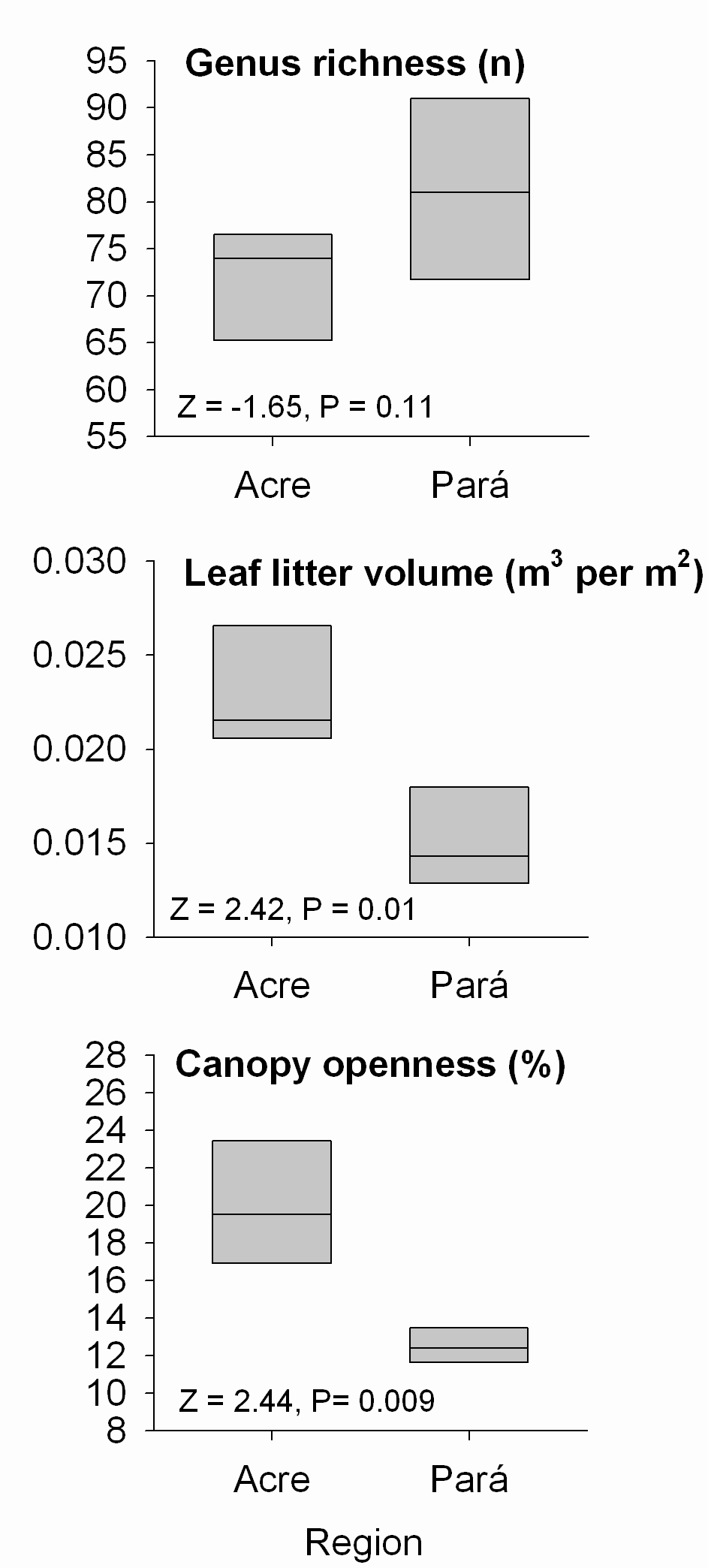


**Figure S3.** Leaf litter depth and canopy openness and the genera richness of stems ≥10 cm DBH along transects placed in unburned forests in Acre (n = 6) and Pará (n = 4) and measured in 2008. Statistics are shown for one-way permutation tests.
