# Supplementary material for: Wildfires in Bamboo-Dominated Amazonian Forest: Impacts on Above-Ground Biomass and Biodiversity
Source: PLoS One. 2012 Mar 9;7(3):e33373. doi: 10.1371/journal.pone.0033373 (PMC3302859; doi:10.1371/journal.pone.0033373)
Supplement: Figure S4 — The mean number of standing dead trees recorded in unburned forests in Pará and Acre. There was no significant difference in any size class, and the overall numbers were almost identical. (DOC) [file pone.0033373.s004.doc]

**Wildfires in bamboo-dominated Amazonian forest: impacts on above-ground biomass and biodiversity**

**Supporting Information Figure S4**


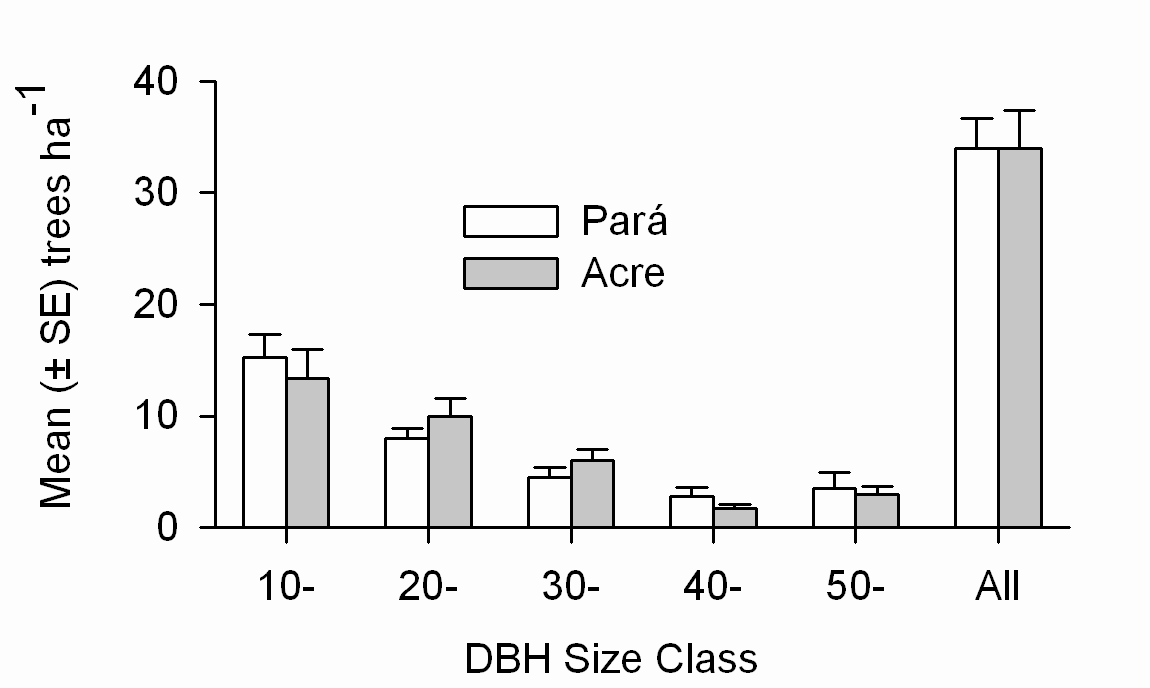


**Figure S4.** The mean number of standing dead trees recorded in unburned forests in Pará and Acre. There was no significant difference in any size class, and the overall numbers were almost identical.
