# Supplementary material for: Wildfires in Bamboo-Dominated Amazonian Forest: Impacts on Above-Ground Biomass and Biodiversity
Source: PLoS One. 2012 Mar 9;7(3):e33373. doi: 10.1371/journal.pone.0033373 (PMC3302859; doi:10.1371/journal.pone.0033373)
Supplement: Figure S5 — Community turnover in birds captured in mist nets in burned and unburned forests, three years after fire in Pará. Data are from [9], but are reanalyzed using the Raup-Crick dissimilarity metric to provide a basis for comparison with the mist net data shown in Figure 6 (main text). (DOC) [file pone.0033373.s005.doc]

**Wildfires in bamboo-dominated Amazonian forest: impacts on above-ground biomass and biodiversity**

**Supporting Information Figure S5**

**Figure S5**. Community turnover in birds captured in mist nets in burned and unburned forests, three years after fire in Pará. Data are from , but are reanalyzed using the Raup-Crick dissimilarity metric to provide a basis for comparison with the mist net data shown in Figure 6 (main text).

1. Barlow J, Peres CA (2004) Avifaunal responses to single and recurrent wildfires in Amazonian forests. Ecological Applications 14: 1358-1373.
